# Publication activities relating to digital teaching and learning in the GMS Journal for Medical Education – a descriptive analysis (1984–2020)

**DOI:** 10.3205/zma001580

**Published:** 2022-11-15

**Authors:** Christin Kleinsorgen, Andrea Baumann, Barbara Braun, Jan Griewatz, Johannes Lang, Holger Lenz, Johanna Mink, Tobias Raupach, Bernd Romeike, Thomas Christian Sauter, Achim Schneider, Daniel Tolks, Inga Hege

**Affiliations:** 1 University of Veterinary Medicine Hannover, Foundation, Centre for E-Learning, Didactics and Educational Research (ZELDA), Hannover, Germany; 2 University of Tübingen, Faculty of Medicine, The Competence Center for University Teaching in Medicine Baden-Württemberg, Faculty, Tübingen, Germany; 3 Medical Faculty Mannheim of the University of Heidelberg, Studies and teaching development, digital teaching, Mannheim, Germany; 4 Justus-Liebig University Gießen, Faculty of Medicine, Division for Study and Teaching, Gießen, Germany; 5 LMU Munich, University Hospital, Institute for Medical Education, Munich, Germany; 6 University Hospital Heidelberg, Department of General Medicine and Health Services Research, Heidelberg, Germany; 7 University Hospital Bonn, Institute for Medical Education, Bonn, Germany; 8 University Medical Center, Academic Dean's Office, Division of Medical Education, Rostock, Germany; 9 University Hospital Bern, Inselspital, Department of Emergency Medicine, Bern, Switzerland; 10 Ulm University, Medical Faculty, Office of the Dean of Studies, Ulm, Germany; 11 Leuphana University Lüneburg, Centre for Applied Health Promotion, Lüneburg, Germany; 12 Bielefeld University, Faculty of Medicine, WG Digital Medicine, Bielefeld, Germany; 13 University of Augsburg, Medical Education Sciences, Augsburg, Germany

**Keywords:** literature review, digital teaching, e-learning, digital learning, digital assessment, GMS Journal for Medical Education

## Abstract

**Aims and objectives:**

Digital teaching, learning and assessment have been part of medical education and continuing education for decades. The objective of this review paper is to highlight developments and perspectives in these areas in the GMS Journal for Medical Education (GMS JME).

**Methodology:**

In the spring of 2020, we conducted a systematic literature search of the Journal for Medical Education (JME) and analysed the articles with regard to different categories such as article type, digital tools used or mode of data collection.

**Results:**

Of the 132 articles analysed, 78 were digital interventions (53 of which were exploratory-descriptive), 28 were project descriptions, 16 were surveys of needs or equipment and 10 were concept papers. About one-third of the studies and project reports each dealt with virtual patients or case-based learning, whereas no articles were published on trends such as serious games or virtual reality. Overall, our analysis shows that in many respects, the studies on digital teaching were more broadly based, especially between 2006 and 2010, after which this trend tended to decline again.

**Conclusions:**

Our analysis shows that publications in the JME consider some key aspects of digital teaching in medical education and continuing education, such as educational videos or virtual patients. The variability of information and methods of presentation advocate the use of guidelines to optimise the quality of scientific papers. Furthermore, clues for future research topics and experimental study designs are identified.

## Zusammenfassung

**Zielsetzung:**

Digitales Lehren, Lernen und Prüfen ist bereits seit Jahrzehnten Bestandteil der medizinischen Aus- und Weiterbildung. Das Ziel dieser Überblicksarbeit ist es, Entwicklungen und Perspektiven in diesen Bereichen im GMS Journal for Medical Education (GMS JME) aufzuzeigen.

**Methodik:**

Im Frühjahr 2020 haben wir eine systematische Literaturrecherche im Journal for Medical Education (JME) durchgeführt und die Artikel hinsichtlich verschiedener Kategorien wie Artikeltyp, eingesetzte digitale Tools oder Modus der Datenerhebung analysiert.

**Ergebnisse:**

Von den 132 analysierten Artikeln waren 78 digitale Interventionen (davon 53 explorativ-deskriptiv), 28 Projektbeschreibungen, 16 Erhebungen zu Bedarfen oder Ausstattungen und 10 Konzeptpapiere. Jeweils etwa ein Drittel der Studien und Projektberichte beschäftigten sich mit virtuellen Patient*innen bzw. fallbasiertem Lernen, während keine Artikel zu Trends wie Serious Games oder virtuellen Realitäten publiziert wurden. Unsere Analyse zeigt insgesamt, dass in vielerlei Hinsicht die Studien zu digitaler Lehre v.a. zwischen 2006 und 2010 breiter gefächert waren, danach war dieser Trend eher wieder rückläufig.

**Schlussfolgerungen:**

Unsere Analyse zeigt, dass Publikationen im JME einige Schwerpunkte der digitalen Lehre in der medizinischen Aus- und Weiterbildung, wie z.B. Lehrfilme oder virtuelle Patient*innen, berücksichtigen. Die Variabilität von Angaben und Darstellungsweisen befürworten den Einsatz von Leitlinien zur Optimierung der Qualität von wissenschaftlichen Arbeiten. Weiterhin werden Anhaltspunkte für zukünftige Forschungsthemen und experimentelle Studiendesigns identifiziert.

## 1. Introduction

Digital teaching and learning are gaining increasing attention, but it is by no means a new approach; they have been known under various terms in university education and continuing education since the 1960s. Alternatively to digital teaching, terms such as e-learning, computer-based learning (CBL) or, since the development of the Internet, web-based training (WBT) have been and are used [1]. In spite of this long period of time, a review shows that there are often quality deficits in the design, data collection and ethical aspects of implementing studies on digital teaching and learning [2]. Since the 1960s, the format of digital teaching has evolved from computer applications to web-based training modules to Massive Open Online Courses (MOOC) [3] or simulations in virtual worlds. Current trends include adaptive learning or learning applications based on Artificial Intelligence (AI), Learning Analytics as well as Big Data [4]. The target groups of digital learning in the health professions have also evolved and now include not only students, trainees, teachers and clinicians, but also patients and relatives [5]. A study by Matthes et al. (2017) already showed the increasing importance of medical education in general with an analysis of articles in the GMS Journal for Medical Education (GMS JME) [6]. However, as this study covered all topics of medical education, no conclusions can be drawn regarding specific topics such as digital learning and teaching.

Therefore, the aim of our analysis was to investigate to what extent and how changes and increasing availability of digital tools and techniques are also reflected in the implementation of studies and curricular use in faculties and schools in JME since the first publication of the journal.

From this, conclusions can be drawn about what the focus is in the publication of research on digital teaching in the German-speaking world and which applications have been less used or researched. Based on this, future directions, further developments and recommendations can be derived.

## 2. Methods

### 2.1. Search strategy

Our search strategy was divided as follows: articles from 1984 (first edition) to 2011 inclusive were searched manually on the JME website due to the lack of indexing. For volumes from 2012 onwards, we were able to use the search function in PubMed with the following query:

((“GMS journal for medical education”[Journal]) OR (“GMS journal for medical education” [Journal])) AND ((e-learning) OR (elearning) or (game) OR (gamification) OR (inverted) OR (flipped) OR (blended) OR (audience response) OR (((digital) OR (app) OR (computer) OR (computer-based) or (online) or (virtual) or (mobile) OR (technology) OR (web-based) OR (multimedia)) AND ((learning) OR (assessment) OR (teaching) OR (education)))).

All articles that met the following inclusion criteria were included in the evaluation:

- Research papers and project reports on the topic of digitisation in education, training and continuing education in health care professions
- Publication date between 1984 (first issue) and May 2020

Excluded were:

- Editorials, abstracts, reviews, position papers and book or film reviews
- Articles not directly related to teaching, learning or assessment, e.g. about purely administrative support

The search and extraction of articles took place in spring 2020.

The assessment of the articles with regard to inclusion or exclusion was carried out independently by two authors. In case of initially divergent assessments, a consensus was reached in a discussion.

#### 2.2. Coding

We developed the categories for coding in an inductive-deductive process. First, categories were developed based on the literature and the authors’ broad experience with digital teaching. The category "type of article" was developed inductively based on the articles, as it became apparent during the coding process that the original division into “study” and “project” was insufficient. After consultation, a coding instruction manual (see attachment 1 ) with examples and explanations was created to instruct the coding. This includes several sections with information on authors, study or project design, digital tools used and type of results (see table 1 (Tab. 1)). The manual was discussed several times, piloted with five articles and subsequently adapted with regard to some category descriptions. The articles were each assigned to two authors for individual coding, then all coding was merged and discrepancies between the two coders were marked. Discrepancies were discussed, and in case of doubt, one or more persons were consulted until consensus was reached. 

## 3. Results

In total through our combined search strategy, we found 221 articles, 89 of which we did not consider for further analysis in the following step due to the exclusion criteria. Thus, 132 articles were included in the further analysis.

### 3.1. Article type and publication year

**Figure 1:**
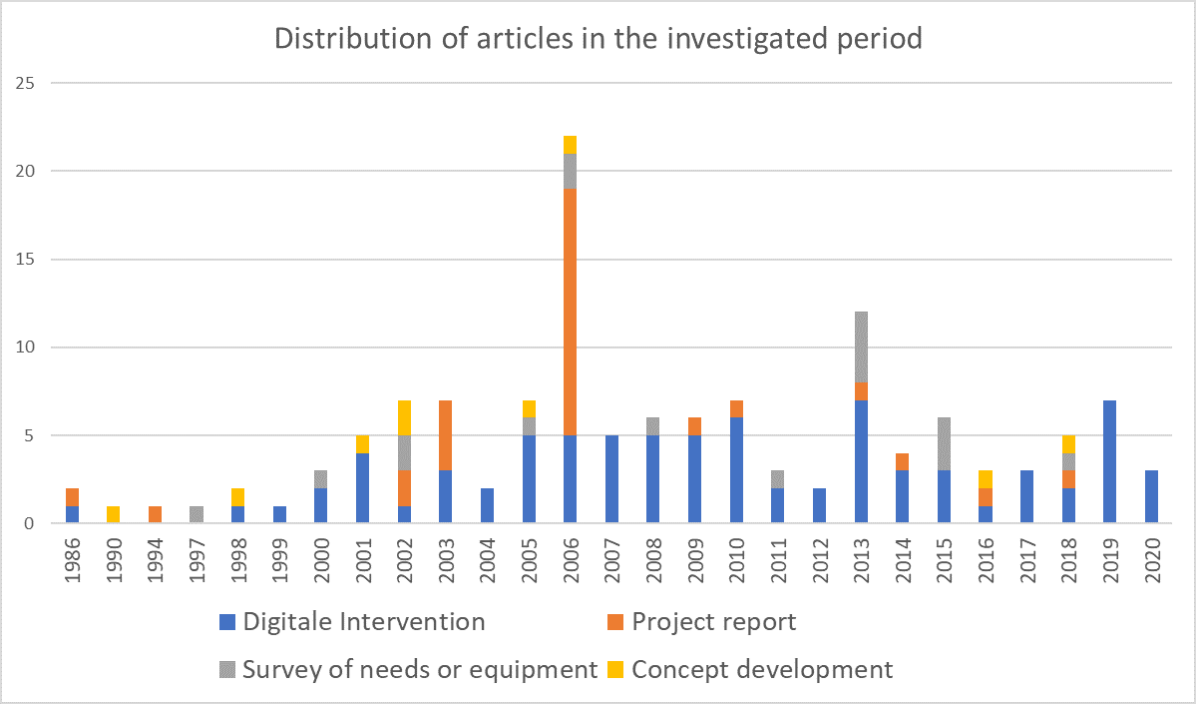
Overview of the articles (N=132) and article types in the investigated period (1984-2020).

Among the 132 articles analysed were 78 studies (59.1%), 28 project descriptions (21.2%), 16 surveys of needs or equipment (12.1%) and 10 concept papers (7.6%). The first article on experiences with the use of an audiovisual program for cardiac screening [7] dates from 1986. The trend in the proportion of studies on digital interventions shows an increase over the studied period (see figure 1 (Fig. 1)).

#### Articles on concept development 

The 10 articles on concept development dealt with considerations on very different topics that were current at the time. The concepts ranged from technical possibilities such as intranet technology [8], computer-based examinations [9] and social media [10], legal and qualitative aspects of digital teaching [11], [12] to considerations of how curricula could be restructured through digitisation [13], [14] or how existing digital tools could be further developed [15].

##### Articles on needs and equipment survey

Eight of the 16 articles (50.0%) on the needs and equipment survey addressed the use of computers and computer labs [16], [17], digital tools in general [18], [19] and specific formats such as wikis [20]. Five articles surveyed current offers and existing equipment for digital teaching [21], [22]. Two articles surveyed a need for digital teaching in each faculty [23], [24] and one article dealt with the survey on the implementation of quality management in the field of digital teaching [25].

##### Project descriptions

The total of 28 project descriptions dealt with both the development of digital tools and the integration and use of digital media at the faculties (n=13 each, 43.3%). Four projects (13.3%) were also or exclusively concerned with the creation of digital content. A trend was not discernible over the studied time period.

##### Studies

Based on the classification of Ringsted et al. (2011) [26], the majority of the 78 studies were exploratory-descriptive ones (n=53, 68.0%), in addition to 14 experimental studies (18.0%) and 11 cohort studies (14.0%). 

Of the 25 experimental or cohort studies, 18 (72.0%) compared different settings. Of these, nine studies (40.9%) compared different digital tools or features; eight studies (36.4%) compared the use of a digital program with an analogue or face-to-face setting; three studies (13.6%) compared a blended learning setting with presence sessions; and two studies had a control group without any intervention. There was no discernible trend over the study period. 

#### 3.2. Methodology & target groups

##### Data collection

Sixty-two of 78 digital studies (79.5%) used questionnaires for data collection, digital platform usage data, and tests or exams were each used by 27 studies (34.6%). Fewer of the studies used interviews (n=9, 11.5%) or focus groups (n=6, 7.7%). One article did not identify how data were collected, one evaluated quality circle meetings [27] and another evaluated written paper feedback [28]. Out of a total of 78 studies, data were collected prospectively in 39 (50.0%) and retrospectively in 37 (47.4%) studies. In two studies, data were obtained both prospectively and retrospectively. There was no discernible trend over the study period.

Among the 16 needs and equipment surveys, questionnaires were used in 11 articles (68.8%), three articles (18.8 %) described an analysis of digital repositories, catalogues or websites, and in two articles, the survey instrument was not clearly specified.

##### Target groups

**Figure 2:**
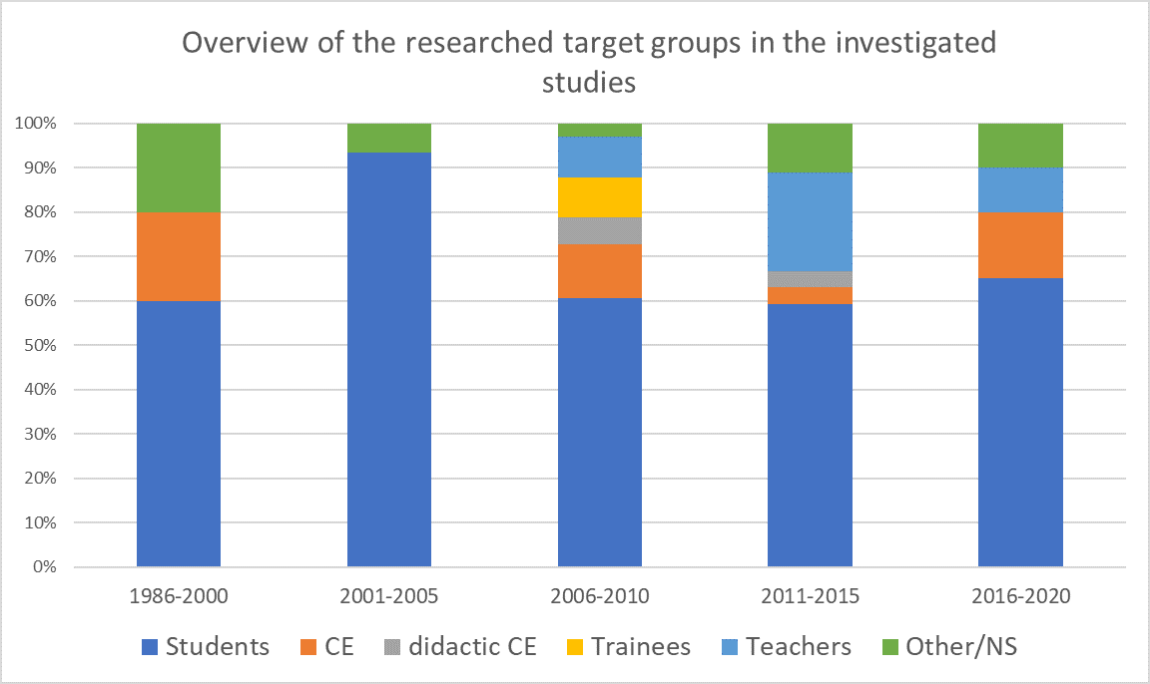
Overview of the target groups which were the focus of research in the studies (n=78): CE=Continuing Education; NS=not specified: in one article, the group of participants was not specified.

Students were the main target group in 66 studies (84.6%). Teachers were addressed as a target group in 11 studies (14.1%), whereas trainees or persons attending a didactic training course were only considered in 3.85 % each (n=3). Other target groups were, for example, parents [29] or prospective students [30]. Figure 2 (Fig. 2) shows the distribution over the study period.

Of the 11 articles on surveys of needs or equipment that were conducted with questionnaires and the two articles with unknown data collection instruments, students were the most frequently surveyed group (n=10, 76.9%), followed by teachers and institutes in three articles each (23.1%), and students, clinicians and participants in continuing education in one article each (7.7%).

In terms of professions, 65 out of the 78 studies (78.3%) involved human medicine, seven involved dentistry (8.4%) and six involved veterinary medicine (7.2%). Interprofessional studies were described in two articles from 2010 and 2011 (2.4%). Other professions included psychology [31] or medical-technical assistants in radiography [32], [33]; there were no studies from nursing. There was no clear trend over the study period; human medicine was the predominant profession, with percentages ranging from 66.7% (2006-2010) to 88.2% (2016-2020) or 100% (1986-2000).

In total, the studies were conducted in 26 different specialised fields or subjects. The most common specialised fields included internal medicine (n=10, 12.2%), studies in interdisciplinary or multidisciplinary settings (n=10, 12.2%) and biochemistry (n=9, 11.0%). Other specialist fields included general medicine, anatomy, surgery as well as cross-cutting areas such as clinical skills, gender medicine and communication training. There was no discernible trend over the study period.

#### 3.3. Study design

Sixty-four (82.1%) of the 78 studies were performed at one single-site, 13 (16.7%) were multi-centric, and in one article (3.2%), the location of study could not be classified. An increase in multi-centric studies was evident over the study period.

**Figure 3:**
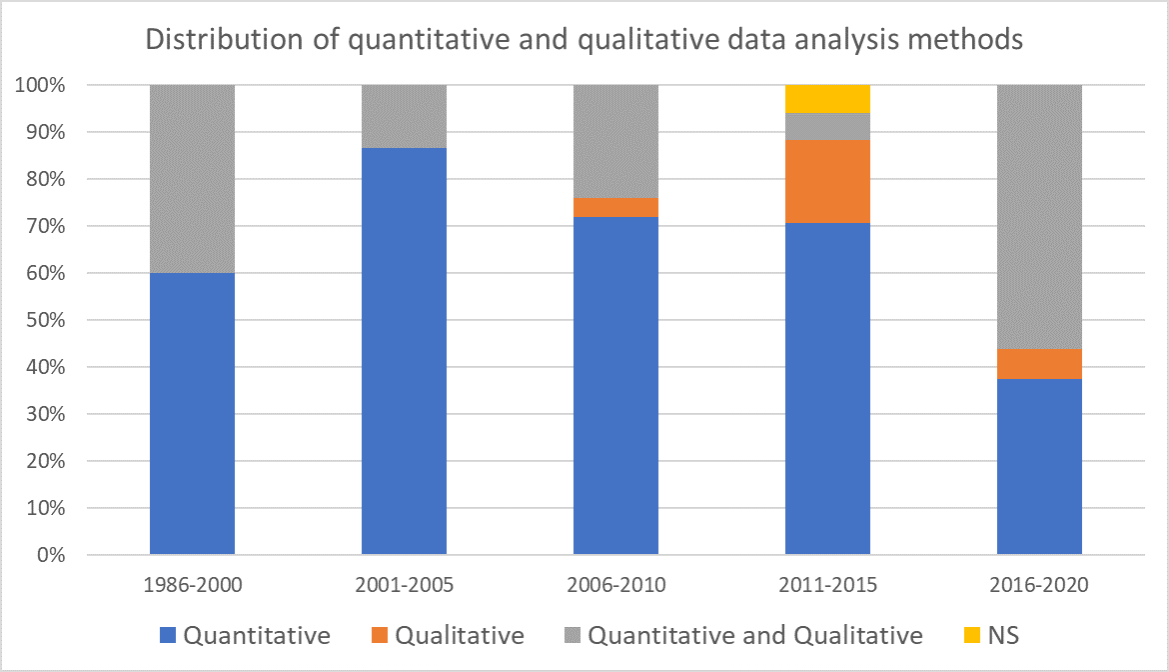
Distribution of quantitative and qualitative data analysis methods used in the studies under review (n=78). NS=not specified

Data evaluation was quantitative in 52 studies (66.7%), qualitative in five studies (6.4%) and both quantitative and qualitative in 20 studies (25.6%). One study could not be classified (1.4%). While quantitative studies predominated until 2005, a qualitative study was published for the first time in 2009 [34], and as of 2016, studies with combined survey instruments predominate (see figure 3 (Fig. 3)). 

In the 16 survey of needs or equipment, data analysis was predominantly quantitative (n=15, 93.8%); only one article described a combination of quantitative and qualitative methods.

##### Kirkpatrick

**Figure 4:**
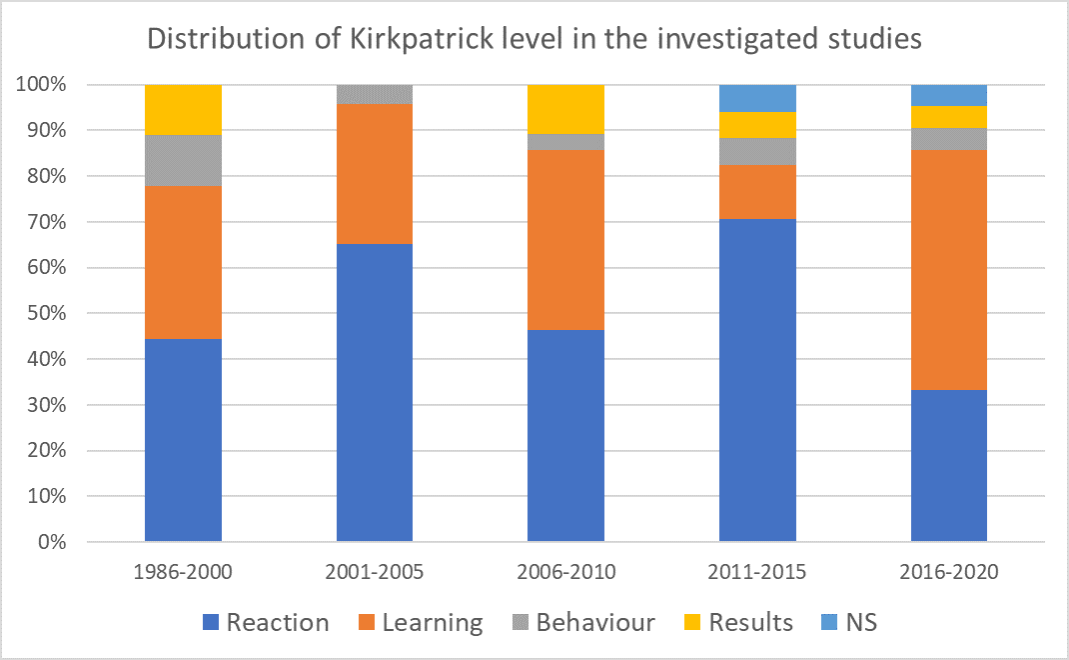
Distribution of Kirkpatrick level [48] in the investigated studies (n=78) (multiple answers possible). NS=not specified

Fifty-one of the 78 study findings (65.4%) were based on the reaction level, capturing, for example, satisfaction with a program, 34 (43.6%) were based on the learning level, five (6.4%) were based on the behavioural level and six (7.7%) were based on the results level. The Kirkpatrick level was not evident in six articles (7.7%). There is no discernible trend over the studied time period; there is a wide variation between the time periods (see figure 4 (Fig. 4)).

#### 3.4. Digital implementation

##### Activities

**Figure 5:**
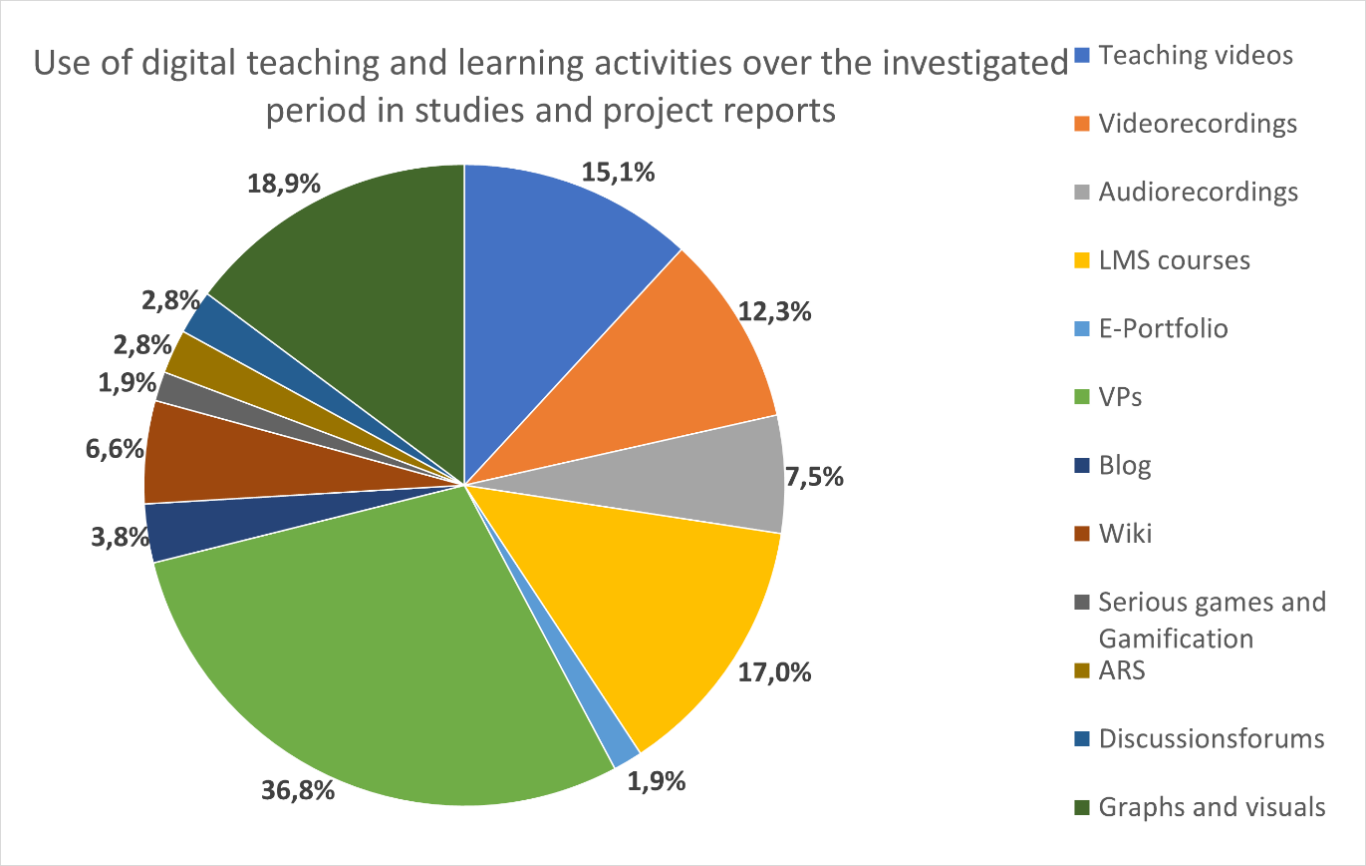
Use of digital teaching and learning activities over the investigated period in studies and project reports (n=106). LMS= learning management system, VPs= virtual patients/case, ARS=audience response system.

The studies most frequently examined virtual patients/cases (VPs) (n=26, 33.3%) and courses in learning management systems (LMS) (n=15, 19.2%). In contrast, no studies were found on serious games, MOOCs or virtual reality (VR) scenarios. Some activities (e.g. instructional videos, video recordings and visual materials) were described rather less frequently, especially up to 2010 and thereafter. Otherwise, no trends are discernible over the studied period (see figure 5 (Fig. 5)).

Among the 28 project descriptions, VPs (n=13, 46.4%) also predominated in individual activities, followed by educational videos (n=8, 28.6%) and video recordings (n=7, 25.0%). A total of 11 projects (39.3%) dealt more cross-cuttingly with e-learning platforms and repositories, for example. There was one project report on a serious game, but no project descriptions on MOOCs, e-portfolios, blogs, virtual reality (VR), gamification and audience response systems (ARS).

##### Study setting

**Figure 6:**
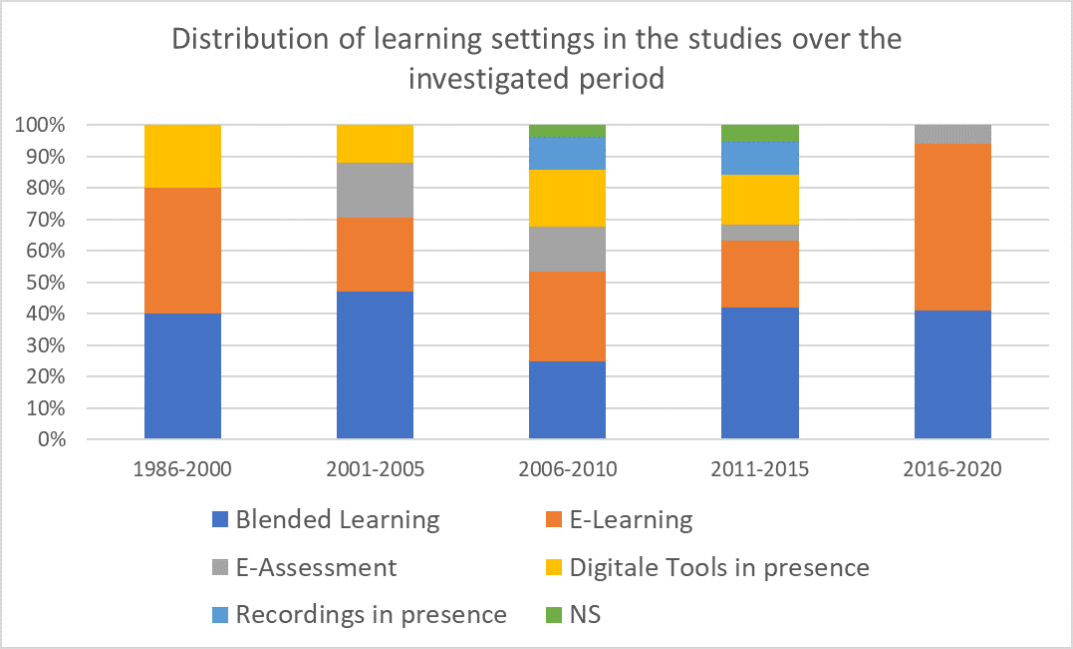
Distribution of learning settings (ICAP) [47] in the studies over the investigated period (n=78) (multiple answers possible) NS=not specified

Most frequently, the studies examined blended learning settings, i.e. a balance between presence and digital teaching (n=32, 41.0%); 27 studies (34.6%) examined pure e-learning settings, 11 (14.1%) examined the use of digital tools or programs in presence teaching and nine (11.5%) dealt with computer-based assessment. Five articles (6.4%) described video recording, e.g. for the purpose of feedback in communication training. Two articles (2.6%) did not identify the setting. Figure 6 (Fig. 6) shows the chronological course; a discernible trend is not evident here.

In terms of access and availability, programs which were available online played a dominant role with 74.4% (n=58), increasing to 100 % since the first online study in 1999. In contrast, offline applications (e.g. in form of CD-ROMs) played an increasingly minor role with 19.2% (n=15), especially since 2011. Studies on mobile devices were also hardly considered, with only 2.6% (n=2). Access was not evident in four articles (5.1%).

The proportion of synchronous and asynchronous study settings was roughly equally distributed over the entire study period, however, with a decrease in synchronous and an increase in asynchronous settings.

##### ICAP Activities

**Figure 7:**
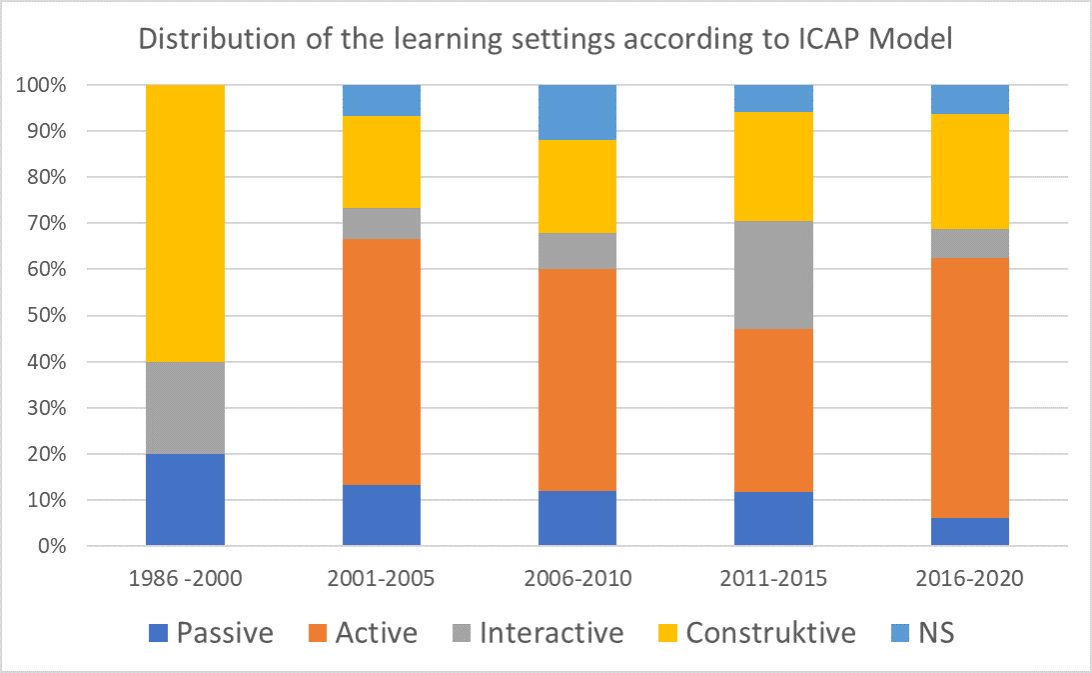
Distribution of learning settings (ICAP) [47] in the studies over the investigated period (n=78). NS=not specified

In the digital studies during the studied time period, active learning settings were most common with 44.9% (n=35), followed by constructive with 24.4% (n=19). The ICAP level was not evident in six articles (7.7%). There was a tendency for passive learning settings to decrease over the studied time period, whereas active ones tended to increase. No trend can be identified for constructive and interactive learning settings (see figure 7 (Fig. 7)).

Fifty-five of the 78 (70.5%) digital studies aimed to teach knowledge and skills, 12 (15.4%) aimed to teach skills and five (6.4%) aimed to teach attitudes; the competence level was not specified in six articles (7.7%). There was no discernible trend over the investigated period.

In addition to the coded aspects, overall it was noticeable when reviewing the articles that it was not always the case that research questions were always clearly formulated, and some studies were not well theoretically grounded.

## 4. Discussion

Against the background of increasing digitisation in medical education and healthcare, we analysed the published articles in the period from the first publication of the JME to May 2020 regarding developments in this area.

Overall, no general increase in articles on digital teaching could be identified in the period under consideration, but rather a continuous increase in studies since 1986 as well as a striking accumulation of project reports in the period between 2006-2010. The latter is probably related to the publication of a conference volume in JME on a symposium on “E-Learning in Medicine” in 2006.

Similarly, as Matthes et al. (2017) demonstrated in an analysis of all JME articles between 2007-2015 [6], exploratory (descriptive) studies also predominate in the topic of digital teaching.

Despite the already available evidence, almost 60% of non-descriptive studies compared digital to non-digital settings or to control groups with no intervention. Cook et al. (2009) argued already in 2009 that further studies comparing digital and traditional learning settings or no intervention do not contribute anything new to the research because digital teaching has already been shown to be as effective as traditional teaching and more effective than no teaching at all [35], [36]. Instead, Cook et al. (2009) see research gaps regarding when digital teaching should be used and how to make it most effective. To this end, more randomised controlled trials examining different digital deployment scenarios in terms of their effectiveness would also be desirable in JME and would advance the state of current research. Even though studies with purely qualitative designs or even studies with qualitative as well as quantitative survey methods increased over the period of investigation, complementary mixed-method designs could actually only rarely be identified [37].

A total of 65.4% of the studies measured the response of the study participants compared to approximately 50% internationally [38], [39], and 43.6% measured the learning outcome by analysing usage data or using knowledge tests. Future studies could focus even more on the behavioural and outcome levels, which have not been given much consideration so far. 

With regard to the target groups or study participants, there were more studies from the non-human medical professions and interprofessional studies, especially in the period 2006-2015, but human medicine predominated overall. Surprisingly, no interprofessional studies on digital teaching were published in the period 2016-2020 despite the special issue “interprofessional education” in 2016. Among the study participants, there was a diversification, especially in the years 2006-2010, with teachers, trainees and participants in didactic and professional development courses. Students were the predominant target group in the JME, both in studies (84.6%) and in surveys of needs or equipment (76.9%). In an international comparison, the focus here in German-speaking countries seems to be more on education; in a review by Curran et al. (2017), only 56% of studies were related to education [36]. Studies show that online learning is also suitable for training interprofessional collaboration and, despite technical challenges, addresses geographical and time barriers, among others, and enables more flexible learning [40], [41], [42]. In the future, we believe that a stronger focus on studies on digital teaching and learning in interprofessional settings and with a greater diversity of study participants would be desirable in order to address this important aspect.

Regarding the digital formats and tools used, the focus of the studies and projects was over 30% on case-based learning/virtual patients. Studies or projects with social media were not considered very much, despite a special issue on this topic in 2013. Also, no studies or project reports on other past and current trends such as MOOCs, serious games or virtual reality learning scenarios were published in the JME. One explanation could be that these topics are published in special journals such as “simulation in healthcare”. Nonetheless, it is also possible that such formats do not (yet) play a major role in medical education in German-speaking countries. One reason could be the comparatively high effort and costs in developing such formats, especially compared to other simulation formats such as virtual patients (VPs) [43]. This could also be the reason why mobile applications have hardly been considered in studies even in recent years. While it can be assumed that most online applications were also playable on mobile devices, specific apps or studies targeting the use of mobile devices were not described.

More than 70% of the studies targeted knowledge transfer, including that through the use of formats such as VPs that are not primarily used for knowledge transfer [44]. Nevertheless, due to the now wide variety and availability of digital formats and tools, skills, collaboration and communication competencies can also be taught through digital and blended learning based teaching.

In conclusion, as also described by Nicoll et al. (2018), some studies lacked or did not clearly describe details such as research questions, theoretical foundation, study population or data collection. This makes study results difficult to interpret or transfer [2]. For future research, we recommend theoretical foundation, e.g. of instructional design frameworks, and description of the study using guidelines such as an extension proposed for simulations by Cheng et al. (2016) [45].

### Limitations 

By limiting our search to one medical didactic journal in the German-speaking area, some relevant articles published for example in medical journals or other international medical didactic journals could certainly not be considered. However, in the analysis, we were also explicitly interested in presenting the developments in JME and drawing conclusions from them.

## 5. Conclusions

Overall, our analysis shows that in many respects, studies on digital teaching were more broadly based, especially between 2006 and 2010, after which this trend tended to decline again. Nonetheless, the articles analysed were submitted before the COVID-19 pandemic, so the resulting changes in the field of digital teaching had not yet been taken into account. We hope that even after the pandemic, many ideas of digital implementation, many of which have already been published as project reports in a COVID-19 special issue [46], will be sustainably implemented, further developed and researched. To this end, we would like our recommendations, based on the analysis, to provide clues for future research topics and experimental study designs. It may also be possible to bring these further to the fore through the publication of special issues on special digital aspects.

## Funding

This Open Access publication was funded by the Deutsche Forschungsgemeinschaft (DFG, German Research Foundation) - 491094227 “Open Access Publication Funding” and the University of Veterinary Medicine Hannover, Foundation.

## Competing interests

The authors declare that they have no competing interests. 

## Supplementary Material

Coding guideline

## Figures and Tables

**Table 1:**
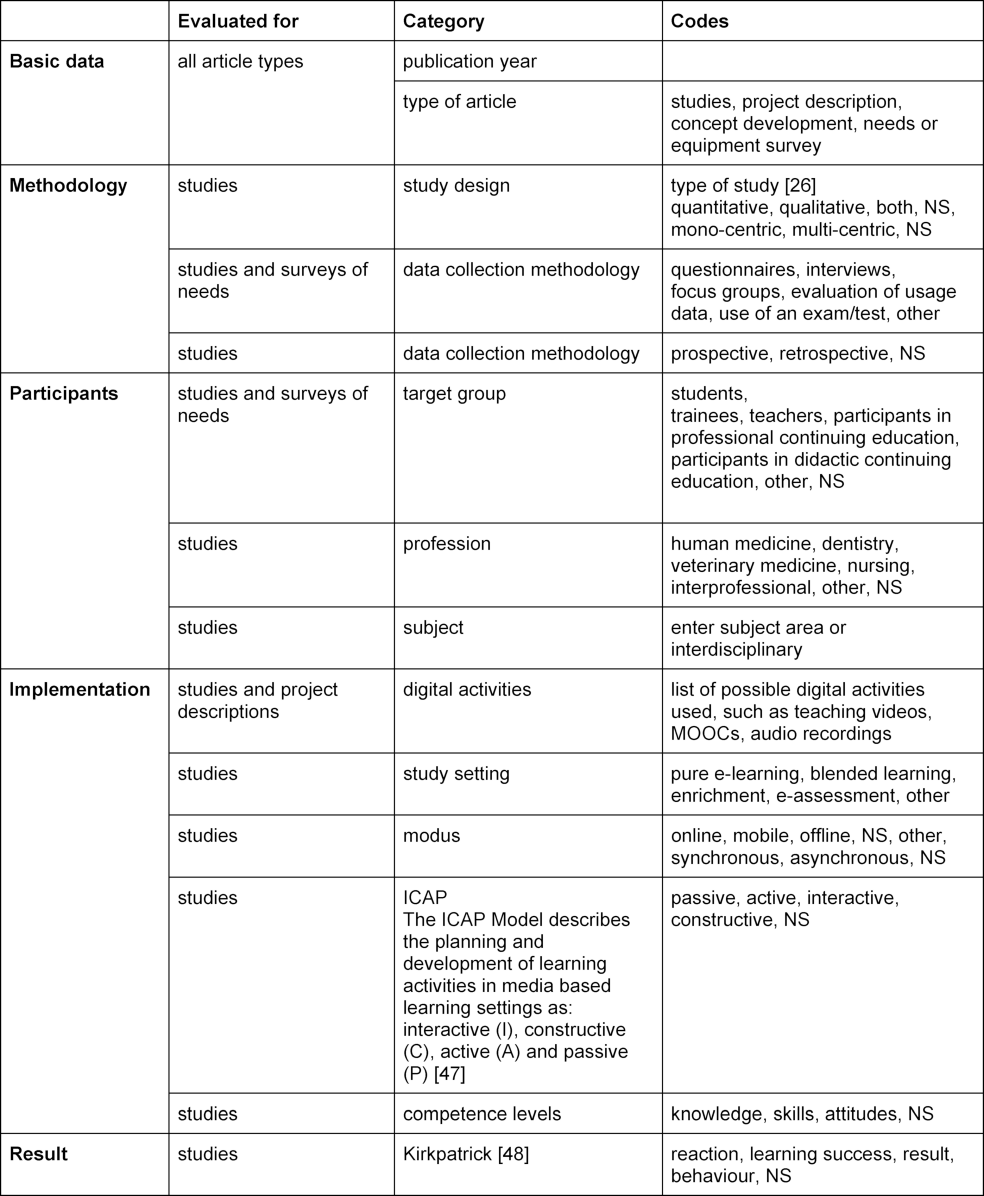
Overview of coding categories; NS=not specified
