# Supplementary material for: Publication activities relating to digital teaching and learning in the GMS Journal for Medical Education – a descriptive analysis (1984–2020)
Source: GMS J Med Educ. 2022 Nov 15;39(5):Doc59. doi: 10.3205/zma001580 (PMC9733476; doi:10.3205/zma001580)
Supplement: Coding guideline [file JME-39-59-s-001.pdf]

Attachment 1: Coding guideline

Article type: project report / study / needs or equipment survey / concept paper / not specified
A study should contain a research question and results, a project report is a pure description of an implementation without evaluation results. In contrast to a project report, a concept paper is not yet implemented and describes a project or a concept of an idea.

First author / last author: Gender of the first and last author, which should be identifiable from the first name. If only 1 author then state only as first author. If unclear, select "not specified". In the case of shared first or last authorship, select "Both" if it is a man and a woman.

Funding (yes/no): is the project/study funded, e.g. by the BMBF, EU, DFG etc. If yes, please specify.

Förderung (Ja/Nein): handelt es sich um ein gefördertes Projekt /Studie z.B. durch BMBF, EU, DFG etc, Falls ja bitte angeben.

Study design

Mono-/Multi-centric/Not specified /Not applicable: Were participants from several institutes/schools/faculties involved in the study? E.g.: a study in several faculties/practices/sites = multi-centric

Qualitative/Quantitative/Both: Is it a study that uses qualitative methods (e.g. content analysis, grounded theory), quantitative methods or both.
A description of the sample in a qualitative study is not a quantitative part, nor is the use of free text fields in a questionnaire without these being explicitly evaluated using qualitative methods and the results presented accordingly, not a qualitative part.

Comparison (selection): If it is a study/project in which group comparisons were carried out, please indicate whether a digital format was compared with a presence format (e.g. paper cases with computer-based cases) or two digital formats (e.g. two different video formats) or a digital format was compared with an analogue format (e.g. when computer cases and paper cases are used once in a face-to-face event).

Study design: (see Ringsted 2011) explorative (descriptive, qualitative, psychometric) / experientiell / Cohort study / translational

Data collection for studies and surveys of needs or equipment: Multiple answers possible:
● **Questionnaires (yes/no):** Were questionnaires (e.g. online, paper) used and evaluated?
● **Interviews (yes/no):** Were interviews (open, structured, etc.) conducted and evaluated with study/project participants?

● **Focus groups (yes/no):** Have focus groups been conducted and evaluated?
● **Evaluation of usage data (yes/no):** Was log data of the users/participants collected and evaluated in any way from a learning platform? Examples: Duration of processing, frequency, scores, but also posts in a discussion forum or wiki entries. The data can be on an individual or aggregated level.
● **Test/Exam (yes/no):** Were for example knowledge tests or a practical exam used to check learning success.
● **Other (free text):** Please describe

Data collection: was the data collected retrospectively, prospectively, or possibly both. prospective: verification of an initial hypothesis with data collected specifically for this purpose vs. retrospective: evaluation of existing data collections after a hypothesis has been formulated.

Study participants: (multiple answers possible)
● **Students (yes/no):** Students in the pre-clinical or clinical section (or in a model course) and Bachelor/Master students
● **Continuing education (yes/no):** continuing education or further training, e.g. for doctors or nurses (e.g. on new diagnostic or therapeutic procedures or similar)
● **Didactic training (yes/no):** Didactic training, e.g. for teachers (e.g. designing lectures or bedside-teaching, etc.) also within the framework of faculty development programs.
● **Training (yes/no):** e.g. trainees at nursing or physiotherapy schools or similar.
● **Lecturers (yes/no)** in their capacity as lecturers (not as participants in a didactic or specialist continuing education course), e.g. if the lecturers were also surveyed as part of a student event.
● **Miscellaneous** (free text)

Profession (selection): from which profession do the participants come? If several, select "interprofessional" (refers to the course of study). If not in the list, please specify under Other (free text).

Subject/subjects (free text): If the study/project relates to a specific subject (e.g. anatomy, internal medicine) or a subject area (e.g. communication, clinical reasoning, etc.), please indicate here. Otherwise enter "Not Specified". If necessary, please list all subjects

Implementation

Activities: Which activities were used / examined, this is primarily about the systems with which the participants learned, not e.g. the evaluation instruments or analysis tools used.

Multiple selection possible:
● **Educational videos (yes/no)** (e.g. training films)
● **Video recordings (yes/no)** (e.g. lecture recordings, including interactive post-processing) with video/screencast and audio
● **Audio recordings (yes/no):** Pure audio recording of e.g. a lecture.

● **MOOC (Yes/No)** (Massive open online course) Course that is openly offered on a classic MOOC platform
● **LMS course(s) (yes/no):** Course(s) in a learning management platform (e.g. moodle, Ilias, Blackboard, etc.)
● **E-Portfolio (yes/no),** use of appropriate software (e.g. Mahara) or use of other software but in the sense of a portfolio of the participants.
● **Virtual patients/cases (yes/no):** computer-based case studies or virtual patients, which are usually interactive, use of appropriate systems if necessary (e.g. CAMPUS, CASUS, d3webTrain, Inmedea,...)
● **Blog (yes/no):** Use of blogs with appropriate software (e.g. WordPress) but also use of blog functionalities from other systems (e.g. LMS)
● **Wiki (yes/no):** Use of wikis either with special systems (e.g. MediaWiki) or also through wiki functionalities of other systems (e.g. in LMS)
● **Serious game (yes/no):** it is a serious game - a digital game that focuses on an entertaining learning experience
● **VR (yes/no):** is it about an activity in the virtual world (e.g. SecondLife)
● **Gamification (yes/no):** Are elements of gamification relevant, e.g. badges,
● **Audio Response System (ARS) (yes/no):** mostly used in face-to-face events, e.g. classic "clickers" or via mobile devices
● **Other (free text):** Please enter here if applicable.

Setting: multiple answers possible (e.g. if different settings were compared with each other)
● **Blended learning:** combination of coordinated face-to-face and computer-based teaching (e.g. inverted classroom)
● **Enrichment:** Use of digital tools (e.g. audio response systems or computer-based learning cases in classroom teaching)
● **Recording:** an event, e.g. on communication training, for later evaluation and feedback
● **pure e-learning (yes/no):** There was only computer-based teaching/learning
● **E-Assessment (yes/no):** computer-based exams (i.e. the examinees took part in digital form, it is not about computer-based exam administration or evaluation
● **Other (free text):** Please describe

Mode: multiple selection possible (e.g. when comparing different modes)
● **online (yes/no):** the learning took place online, i.e. an internet connection is required (e.g. websites, online learning programs, wikis, etc...)
● **mobile (yes/no):** explicit information about the use of software via mobile phone or tablet
● **offline (yes/no):** Activities work without an Internet connection, e.g. programs on CD-ROM
● **Synchronicity:** Do the activities take place synchronously or asynchronously. This is about the setting of the study, not what the program is for.
● **Other (free text):** please describe if necessary

ICAP: See https://digitales- Klassenzimmer.org/icap-modell/ or https://www.schoology.com/blog/icap-framework-connecting-learning-learning-outcomes

Competence Level: Is this an assessment of knowledge, skills, or attitudes. select Not specified or Not Applicable if necessary.

Results

Kirkpatrick Level: (multiple answers possible) Description see https://educationaltechnology.net/kirkpatrick-model-four-levels-learning-evaluation/
to be considered if measured objectively in the intervention, i.e. self-assessment of learning success would be "reaction", and in shown in the results. If several levels apply, please list them all.
